# Three-dimensional ultrastructure of capillary endothelial glycocalyx under normal and experimental endotoxemic conditions

**DOI:** 10.1186/s13054-017-1841-8

**Published:** 2017-10-23

**Authors:** Hideshi Okada, Genzou Takemura, Kodai Suzuki, Kazumasa Oda, Chihiro Takada, Yasuaki Hotta, Nagisa Miyazaki, Akiko Tsujimoto, Isamu Muraki, Yoshiaki Ando, Ryogen Zaikokuji, Atsumu Matsumoto, Hiroki Kitagaki, Yuto Tamaoki, Takahiro Usui, Tomoaki Doi, Takahiro Yoshida, Shozo Yoshida, Hiroaki Ushikoshi, Izumi Toyoda, Shinji Ogura

**Affiliations:** 1 Department of Emergency and Disaster Medicine, Gifu University Graduate School of Medicine, 1-1 Yanagido, Gifu, 501-1194 Japan; 2 Department of Internal Medicine, Asahi University School of Dentistry, Mizuho, Japan; 3 Research Institute for Biotechnology, Asahi University School of Dentistry, Mizuho, Japan; 4 Laboratory of Molecular Biology, Department of Biofunctional Analysis, Gifu Pharmaceutical University, Gifu, Japan

**Keywords:** Endothelial cell, Glycocalyx, Ultrastructure, Vascular endothelial injury, Sepsis, Capillary

## Abstract

**Background:**

Sugar-protein glycocalyx coats healthy endothelium, but its ultrastructure is not well described. Our aim was to determine the three-dimensional ultrastructure of capillary endothelial glycocalyx in the heart, kidney, and liver, where capillaries are, respectively, continuous, fenestrated, and sinusoidal.

**Methods:**

Tissue samples were processed with lanthanum-containing alkaline fixative, which preserves the structure of glycocalyx.

**Results:**

Scanning and transmission electron microscopy revealed that the endothelial glycocalyx layer in continuous and fenestrated capillaries was substantially thicker than in sinusoids. In the heart, the endothelial glycocalyx presented as moss- or broccoli-like and covered the entire luminal endothelial cell surface. In the kidney, the glycocalyx appeared to nearly occlude the endothelial pores of the fenestrated capillaries and was also present on the surface of the renal podocytes. In sinusoids of the liver, glycocalyx covered not only the luminal side but also the opposite side, facing the space of Disse. In a mouse lipopolysaccharide-induced experimental endotoxemia model, the capillary endothelial glycocalyx was severely disrupted; that is, it appeared to be peeling off the cells and clumping. Serum concentrations of syndecan-1, a marker of glycocalyx damage, were significantly increased 24 h after administration of lipopolysaccharide.

**Conclusions:**

In the present study, we visualized the three-dimensional ultrastructure of endothelial glycocalyx in healthy continuous, fenestrated, and sinusoidal capillaries, and we also showed their disruption under experimental endotoxemic conditions. The latter may provide a morphological basis for the microvascular endothelial dysfunction associated with septic injury to organs.

**Electronic supplementary material:**

The online version of this article (doi:10.1186/s13054-017-1841-8) contains supplementary material, which is available to authorized users.

## Background

The sugar-protein glycocalyx coats all healthy vascular endothelium [1–3] and plays a key role in microvascular and endothelial physiology through its influence on the regulation of microvascular tone and endothelial permeability, maintenance of an oncotic gradient across the endothelial barrier, regulation of adhesion/migration of leukocytes, and inhibition of intravascular thrombosis [4–8]. Components of glycocalyx include cell-bound proteoglycans, glycosaminoglycan side chains, and sialoproteins [9–11]. Proteoglycans consist of a core protein, such as a syndecan family protein, to which glycosaminoglycan is linked. Because glycosaminoglycan side chains contain a high density of negative charges, electrostatic repulsion drives albumin away from the vessel wall, toward the center of the lumen [12].

Endothelial cell structures are specific for each organ and include at least three types of capillaries: continuous, fenestrated, and sinusoidal [13–15]. Continuous capillaries are characterized by the presence of an uninterrupted endothelium with a continuous basal lamina. This type is found in muscle tissues, heart, lung, brain, and other organs. Fenestrated capillaries are found in the renal glomeruli and endocrine glands, among other tissues. They are characterized by the presence of circular fenestrae or pores that penetrate the endothelium. Sinusoids are found in the liver and hematopoietic organs such as the bone marrow and the spleen. Sinusoidal capillaries are a special type of open-pore capillary also known as discontinuous capillaries, which have larger openings with diameters of 30–40 μm in the endothelium. Given the structural and functional differences among the endothelium types, one could speculate that the morphology of glycocalyx would also vary among the different types of endothelial cells.

The endothelial glycocalyx has matrix properties and restricts larger macromolecules to the vessel lumen, which called into question the conventional theory that simple filtration is regulated through variable gaps between the cells, as stated in the Starling Principle of transvascular fluid dynamics [16]. However, the revised Starling Principle suggests Starling forces are only applied across the endothelial glycocalyx as a molecular sieve for plasma proteins [17, 18]. In fact, the hydraulic permeability rises dramatically when the endothelial glycocalyx is experimentally removed [19]. The endothelial glycocalyx is reportedly damaged under stress conditions such as sepsis [20]. Diffuse and persistent alterations in the glycocalyx are linked to widespread endothelial dysfunction, altered permeability, and impaired oxygen and nutrient delivery to cells [8]. However, there have been few reports directly examining the morphology of the glycocalyx in each capillary type. In the present study, therefore, we investigated the three-dimensional ultrastructure of vascular endothelial glycocalyx in the heart, kidney, and liver under normal and pathological conditions.

## Methods

### In vivo animal studies

After starvation for 16 h, 10-week-old male mice were intraperitoneally administered lipopolysaccharide (LPS, 20 mg/kg; Sigma-Aldrich, St. Louis, MO, USA). Forty-eight hours after LPS administration, the survival rate was determined. Blood was then collected from the ophthalmic artery, after which the mice were killed, and heart, liver, and kidney specimens were obtained.

### Electron microscopy

To detect endothelial glycocalyx using electron microscopy [21], mice were anesthetized and perfused with a solution composed of 2% glutaraldehyde, 2% sucrose, 0.1 M sodium cacodylate buffer (pH 7.3), and 2% lanthanum nitrate through a cannula placed in the left ventricle 48 h after LPS administration [22]. Before perfusion, an incision was made in the right atrial appendage, and the neck was ligated with a silk suture. In addition, a perfusion pump was used for injection at a steady rate of 1 ml/minute.

Thereafter, the left ventricle, liver, and kidney were harvested and diced. Three or four pieces of approximately 1 mm^3^ each were immersed in the perfusion solution for 2 h for fixation and then soaked overnight in a solution without glutaraldehyde before being washed in alkaline (0.03 mol/L NaOH) sucrose (2%) solution. The specimens were then dehydrated through a graded ethanol series.

The frozen fracture method was used to prepare samples for examination using scanning electron microscopy (SEM). Each sample was laid on an iron plate chilled with liquid nitrogen, and ethanol was sprinkled onto it. Once the ethanol was frozen, the sample was fractured using a chisel such that it was not touched directly. The samples were then incubated in *tert*-butyl alcohol at room temperature. After the *tert*-butyl alcohol had solidified, it was freeze-dried, and the specimens were examined using SEM (S-4500; Hitachi, Tokyo, Japan). In addition, for further elemental analysis of each sample, energy-dispersive X-ray spectroscopy was performed under SEM.

To prepare samples for transmission electron microscopy (TEM), each specimen was embedded in epoxy resin. Ultrathin sections (90 nm) stained with uranyl acetate and lead citrate were then examined using TEM (HT-7700; Hitachi). For usual electron microscopy, 2.5% glutaraldehyde in 0.1 mol/L phosphate buffer (pH 7.4) was used instead of perfusion buffer as described above.

### Measurement of syndecan-1 in the plasma

Following LPS administration to mice, plasma concentrations of syndecan-1 were measured (*n* = 5) using an enzyme-linked immunosorbent assay (860.090.192; Diaclone, Besancon Cedex, France).

### Quantitative assessments of the endothelial wall thickness

Quantitative assessments of the endothelial wall thickness were performed on six randomly chosen capillary vessels in TEM images using ImageJ software (National Institutes of Health, Bethesda, MD, USA). The average thickness of the endothelial wall was found by measuring at five points of the endothelial capillary wall, except for the nuclear part.

### Quantitative assessments of the endothelial glycocalyx area

Quantitative assessments of the endothelial glycocalyx occupation area of the capillary lumen area were performed on six randomly chosen capillary vessels in TEM images using ImageJ software.

### Statistical analysis

Values are shown as the mean ± SE. Survival was analyzed using the Kaplan-Meier method with the log-rank Cox-Mantel method. The significance of differences was evaluated using *t* tests. *p* < 0.05 was considered significant.

## Results

### Glycocalyx in continuous capillaries

**Fig. 1:**
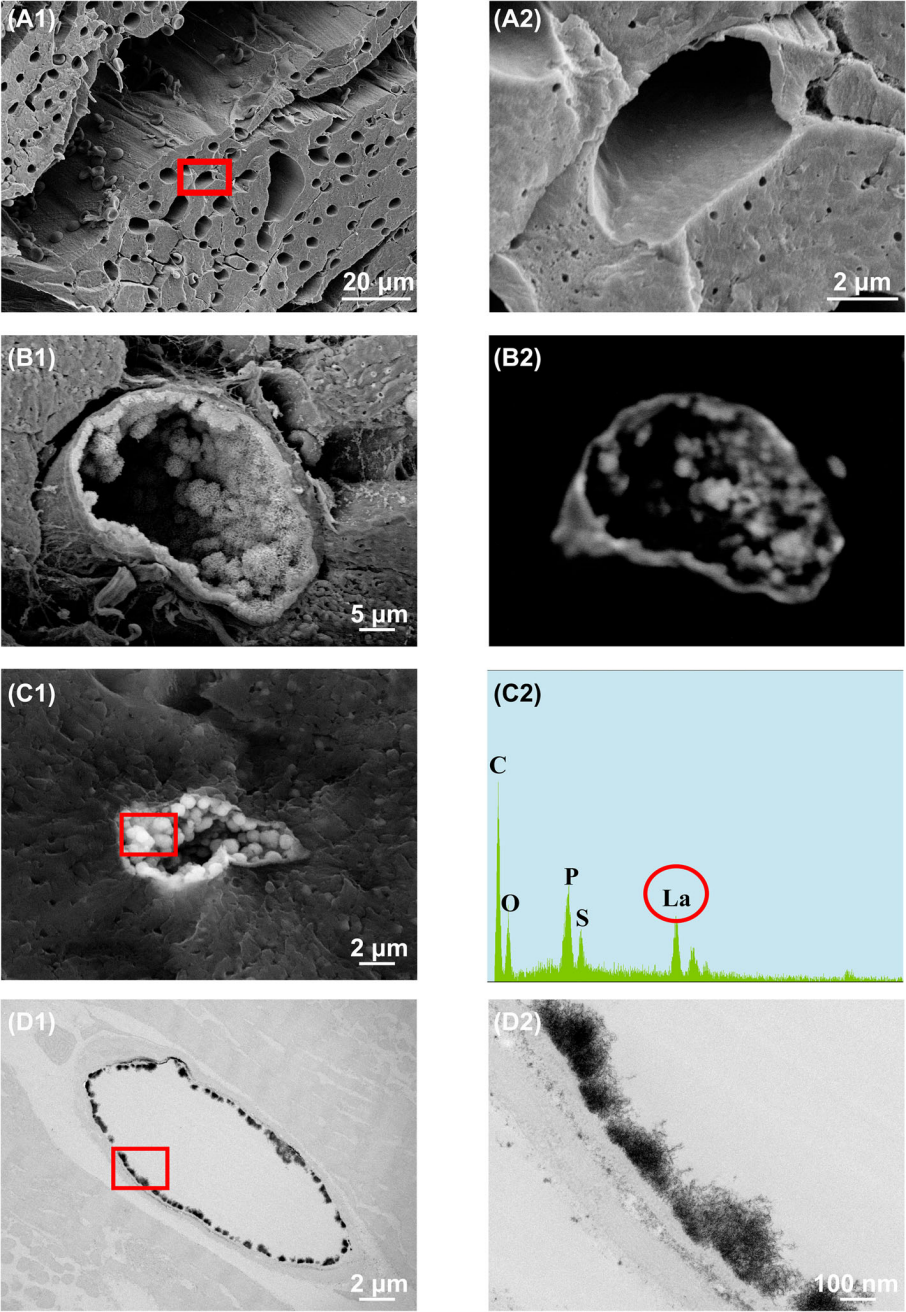
Scanning and transmission electron microscopy showing glycocalyx of continuous capillaries in the heart under normal conditions. **a1** Cardiac capillary without lanthanum nitrate staining. **a2** Expanded view of the area within the *red rectangle* square in (**a1**). Continuous capillaries in the heart have a continuous thin basement membrane. **b1** Cardiac capillary with lanthanum nitrate staining. The endothelial glycocalyx, which is the bush-like structure, can be seen on the surface of the vascular endothelium. **b2** Backscattered electron microscopic image of the same specimen as in (**b1**). The location of backscattered electrons is consistent with the bush-like structure. **c1** Energy-dispersive spectroscopic image of a cardiac capillary stained with lanthanum nitrate. **c2** Ingredient analysis of the area within the *red rectangle* in (**c1**). The bush-like structure includes lanthanum, indicating this structure is the endothelial glycocalyx. *C* Carbon, *O* Oxygen, *P* Phosphorus, *S* Silicon, *La* Lanthanum. **d1** Transmission electron microscopic imaging of cardiac capillary with lanthanum nitrate staining. **d2** Expanded view of the area within the *red rectangle* in (**d1**). The endothelial glycocalyx can also be seen on the surface of the vascular endothelium

Capillaries in the heart are classified as continuous. Standard SEM examination of the luminal side of the cardiac capillary endothelium showed intracellular tight junctions but no transcellular perforations; also undetected was the endothelial glycocalyx (Fig. 1a1 and a2). However, lanthanum nitrate staining revealed moss-like or broccoli-like structures on the endothelial cells, which we suspected were endothelial glycocalyx (Fig. 1b1). To confirm those structures were, in fact, endothelial glycocalyx, we used the backscattered electron method under SEM (Fig. 1b2). The detected backscattered high-energy electrons that rebounded from the sample surface indicated the presence of metals in the sample. The location of backscattered electrons was consistent with a bush-like structure, suggesting the structure was endothelial glycocalyx stained with lanthanum nitrate. For further confirmation, we performed an elemental analysis of this structure using energy-dispersive X-ray spectroscopy (Fig. 1c1). Spectroscopic analysis showed the structure contained lanthanum as well as carbon, oxygen, and phosphorus (Fig. 1C2), which indicates that the structure was glycocalyx. In addition, TEM confirmed the presence of bush-like structures on the surface of the endothelial cells (Fig. 1d1 and d2). The percentage of endothelial glycocalyx area in capillaries was 13.6 ± 2.0%.

### Glycocalyx in fenestrated capillaries

**Fig. 2:**
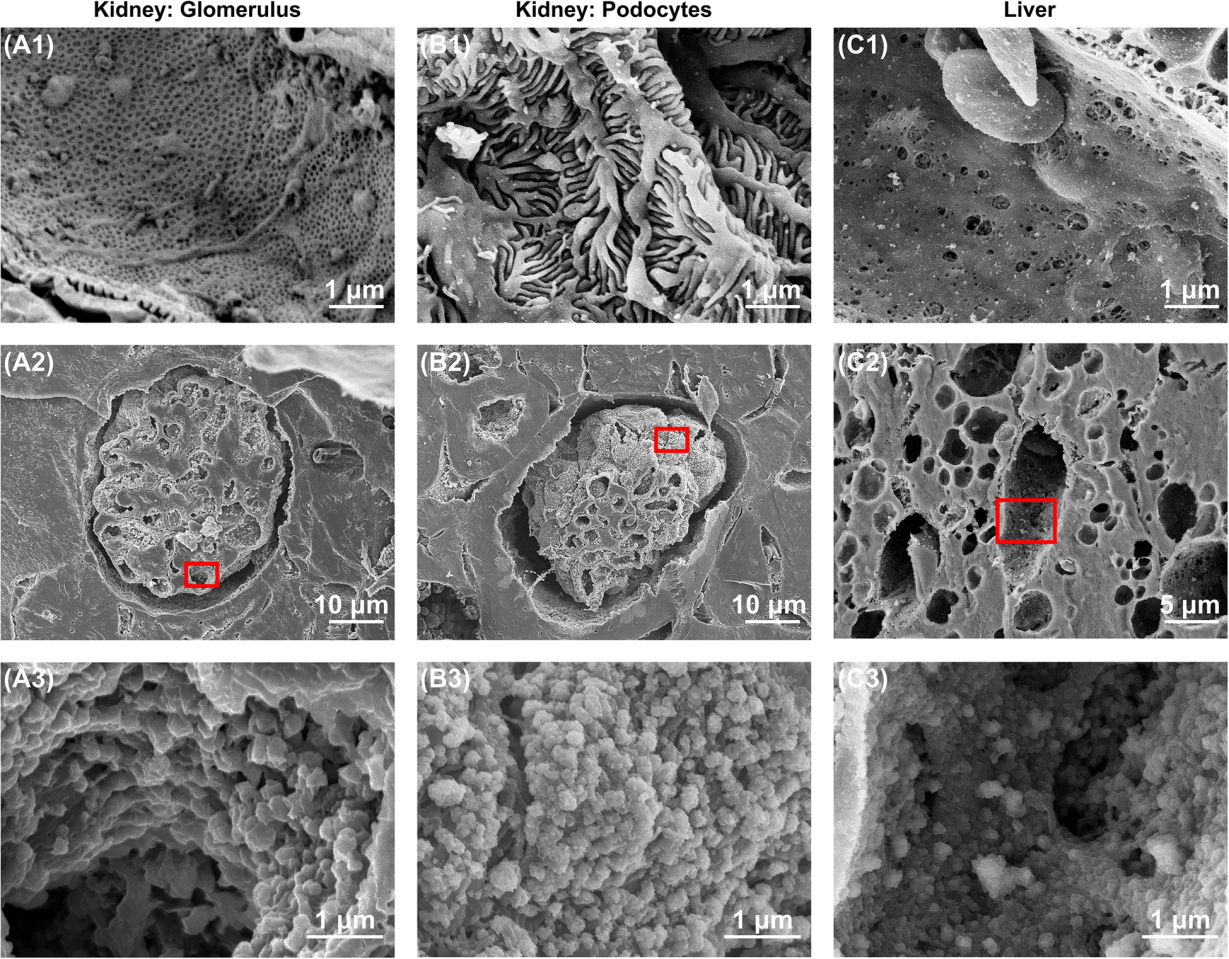
Scanning electron microscopy showing glycocalyx in fenestrated capillaries of the kidney and sinusoids of the liver under normal conditions. **a** Ultrastructure of glomerular capillaries under normal conditions. **a1** Fenestrated capillary without lanthanum nitrate staining. Small pores are present on the surface of the endothelial cells. **a2**, **a3** Lanthanum nitrate staining to visualize endothelial glycocalyx. **a3** Expanded view of the area within the *red rectangle* in (**a2**). Endothelial glycocalyx covers the surface of glomerular capillaries. **b** Ultrastructure of podocytes on the outer surface of the glomerulus under normal condition. **b1** Podocytes without lanthanum nitrate staining. Many podocytes firmly intertwine with each other to form a meshwork. **b2**, **b3** Glycocalyx on podocytes visualized by lanthanum nitrate staining. **b3** Expanded view of the area within the *red rectangle* in (**b2**). Glycocalyx overlays the surface of podocytes. **c** Ultrastructure of hepatic sinusoids under normal conditions. **c1** Sinusoid without lanthanum nitrate staining. Sinusoids in liver are open-pore capillaries. **c2**, **c3** Visualized glycocalyx in sinusoids. **c3** Expanded view of the area within the *red rectangle* in (**c2**). The endothelial glycocalyx in sinusoids does not overlay the open fenestrations but is also present in the space of Disse

**Fig. 3:**
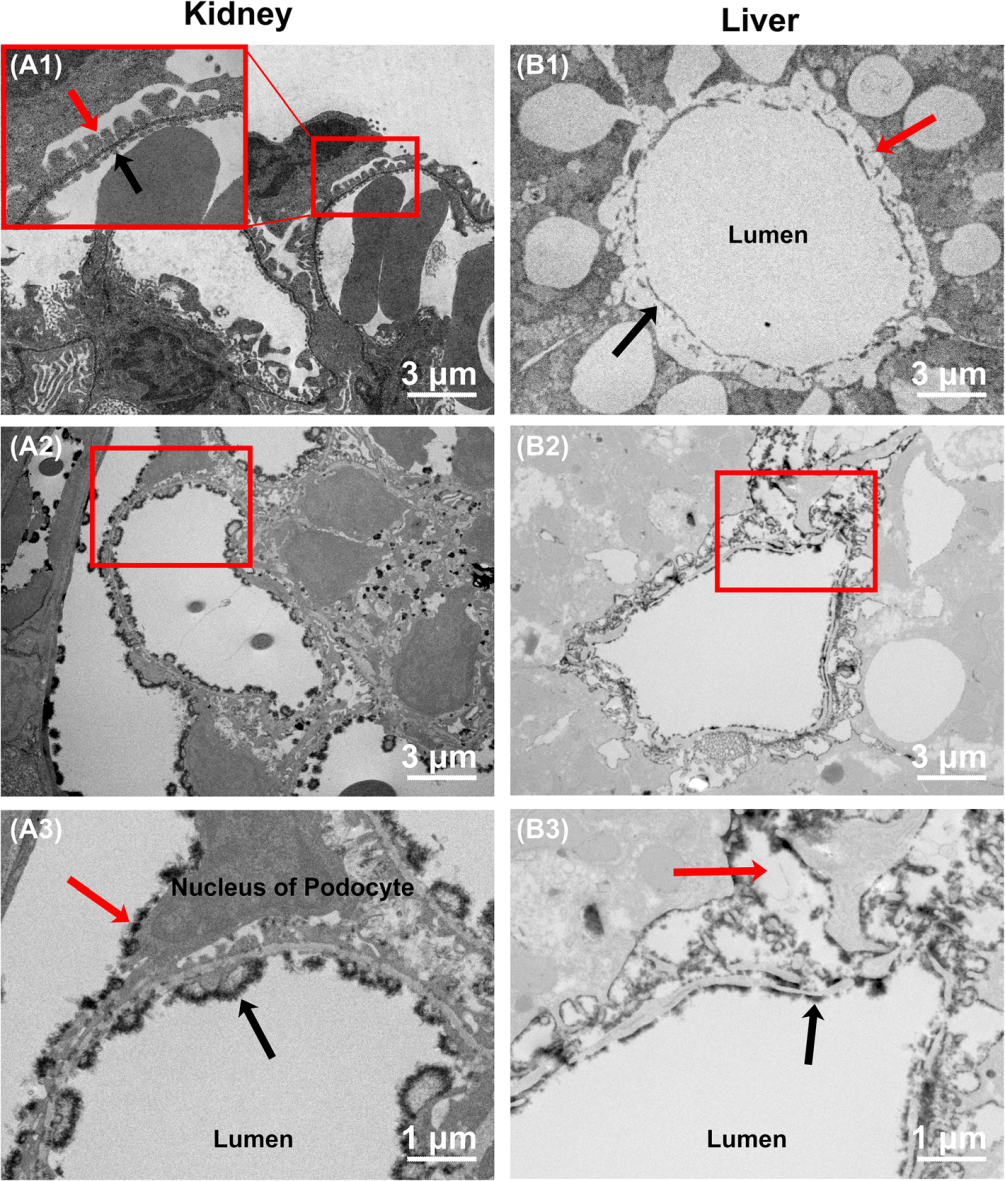
Transmission electron microscopy showing glycocalyx in glomerular fenestrated capillaries and hepatic sinusoids under normal conditions. **a** Ultrastructure of glomerular capillaries under normal conditions. **a1** Glomerular capillary without lanthanum nitrate staining. The healthy glomerular endothelium is composed of three layers, including endothelial cells (*black arrow*), as well as basement membrane and podocytes (*red arrow*), which are bound with each other. **a2**, **a3** Lanthanum nitrate staining to visualize glycocalyx. **a3** Expanded view of the area within the *red rectangle* in (**a2**). Glycocalyx is present on the surface of glomerular capillaries and podocytes. **b** Ultrastructure of hepatic sinusoids under normal conditions. **b1** Sinusoid without lanthanum nitrate staining. The sinusoid is composed of discontinuous flat endothelial cells (*black arrow*) and has large pores. The space of Disse is situated under the endothelium (*red arrow*). **b2**, **b3** Visualized glycocalyx in sinusoids. **b3** Expanded view of the area within the *red rectangle* in (**b2**). The endothelial glycocalyx layer of sinusoids is thin (*black arrow*) and is also present in the space of Disse (*red arrow*)

The endothelial cells comprising the fenestrated capillaries found in renal glomeruli have pores that allow small molecules to penetrate but limit protein diffusion. Using SEM without lanthanum nitrate staining, we readily detected the pores in the glomerular endothelium (Fig. 2a1). In addition, the backscattered electron method confirmed the presence of endothelial glycocalyx covering the luminal surface of the glomerular capillaries (Additional file 1: Figure S1a). SEM with lanthanum nitrate staining showed that the pores were narrowed by glycocalyx such that they were nearly occluded (Fig. 2a2 and a3). In addition, TEM with lanthanum nitrate fixation showed that the endothelial glycocalyx layer lined the open fenestrations and covered the surface of the podocytes (Fig. 3a2 and a3). The percentage of endothelial glycocalyx area in capillaries was 16.7 ± 1.8%.

### Glycocalyx of sinusoids

Sinusoids in the liver form large and irregularly anastomosing structures. The endothelial cells comprising the sinusoid wall are flattened and have no basement membrane (Figs. 2c1 and 3b1). Backscattered electrons were detected from the sinusoidal capillaries in lanthanum nitrate-stained specimens (Additional file 1: Figure S1c). The endothelial glycocalyx of sinusoids did not occlude the open fenestrations, and the height of the glycocalyx was less than in continuous and fenestrated capillaries (Figs. 2c3 and 3b3). In addition, TEM revealed the presence of glycocalyx around the endothelial cells, not only on the luminal side but also on the side facing the space of Disse. The percentage of endothelial glycocalyx area in capillaries was 3.7 ± 0.3%.

### Glycocalyx under septic vasculitis conditions

**Fig. 4:**
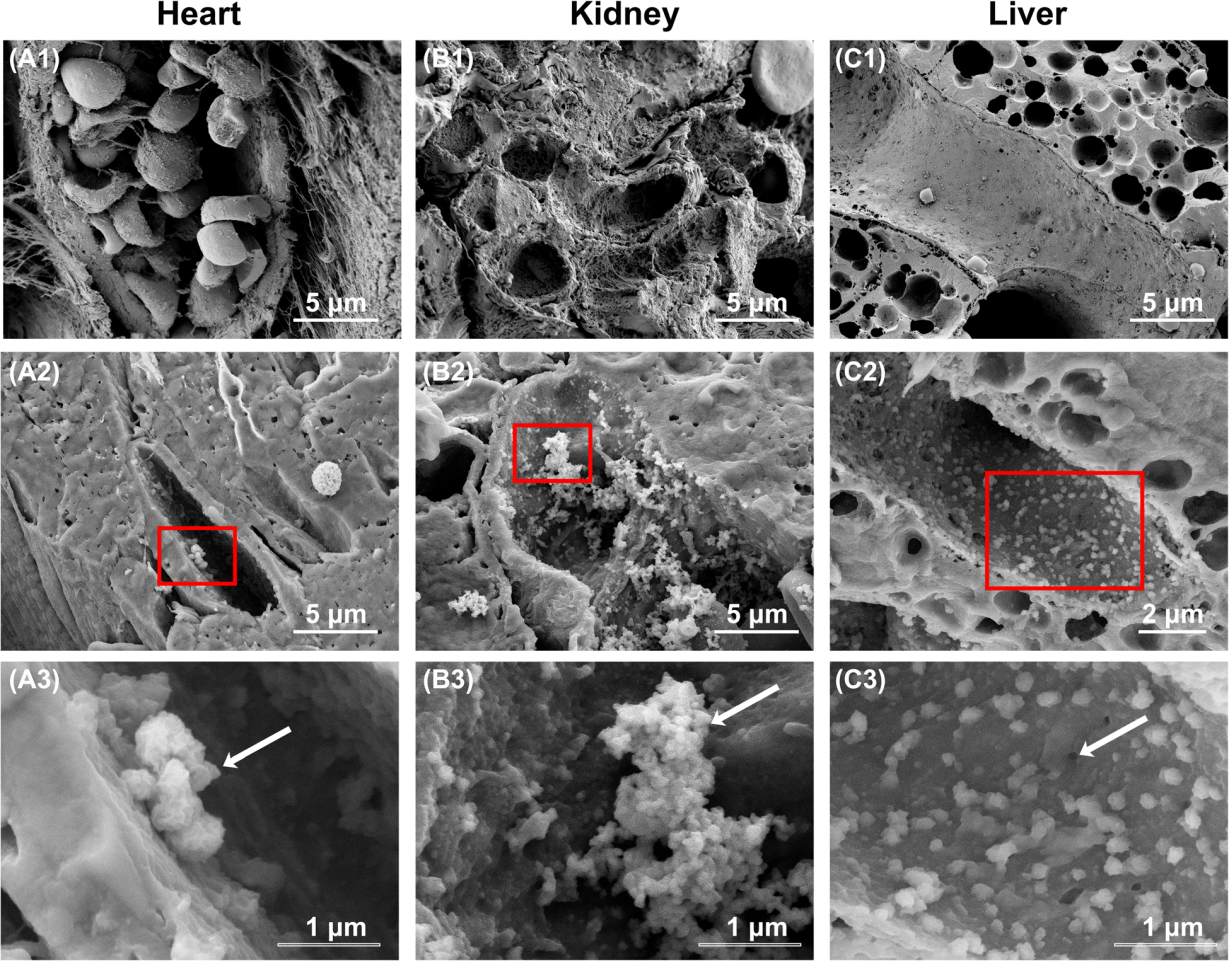
Scanning electron microscopy showing glycocalyx in continuous, fenestrated, and sinusoidal capillaries under septic conditions. **a** Ultrastructure of continuous capillaries in the heart under septic conditions. **a1** Continuous capillary without lanthanum nitrate staining. Thickening of the endothelial wall is presumed to be due to edematous changes related to inflammation. **a2**, **a3** Lanthanum nitrate staining to visualize the endothelial glycocalyx. **a3** Expanded view of the area within the *red rectangle* in (**a2**). The endothelial glycocalyx is peeled away from the surface of endothelial cells, and the residue is found inside the vascular lumen (*white arrow*). **b** Ultrastructure of glomerular capillaries under septic conditions. **b1** Fenestrated capillary without lanthanum nitrate staining. Destruction of the small pore structure is observable. In addition, the endothelial wall appears edematous. **b2**, **b3** Lanthanum nitrate staining to visualize the endothelial glycocalyx. **b3** Expanded view of the area within the *red rectangle* in (**b2**). Glycocalyx is cast off from the endothelial cells, and the residue of it exists inside the vascular lumen (*white arrow*). **c** Ultrastructure of hepatic sinusoids under septic conditions. **c1** Sinusoid without lanthanum nitrate staining. The large pores are nearly completely occluded (*white arrow*). **c2**, **c3** Visualized glycocalyx within sinusoids. **c3** Expanded view of the area within the *red rectangle* in (**c2**). The sinusoidal endothelial glycocalyx is peeled away from the endothelial cells, and the residue is present inside the vascular lumen (*white arrow*)

**Fig. 5:**
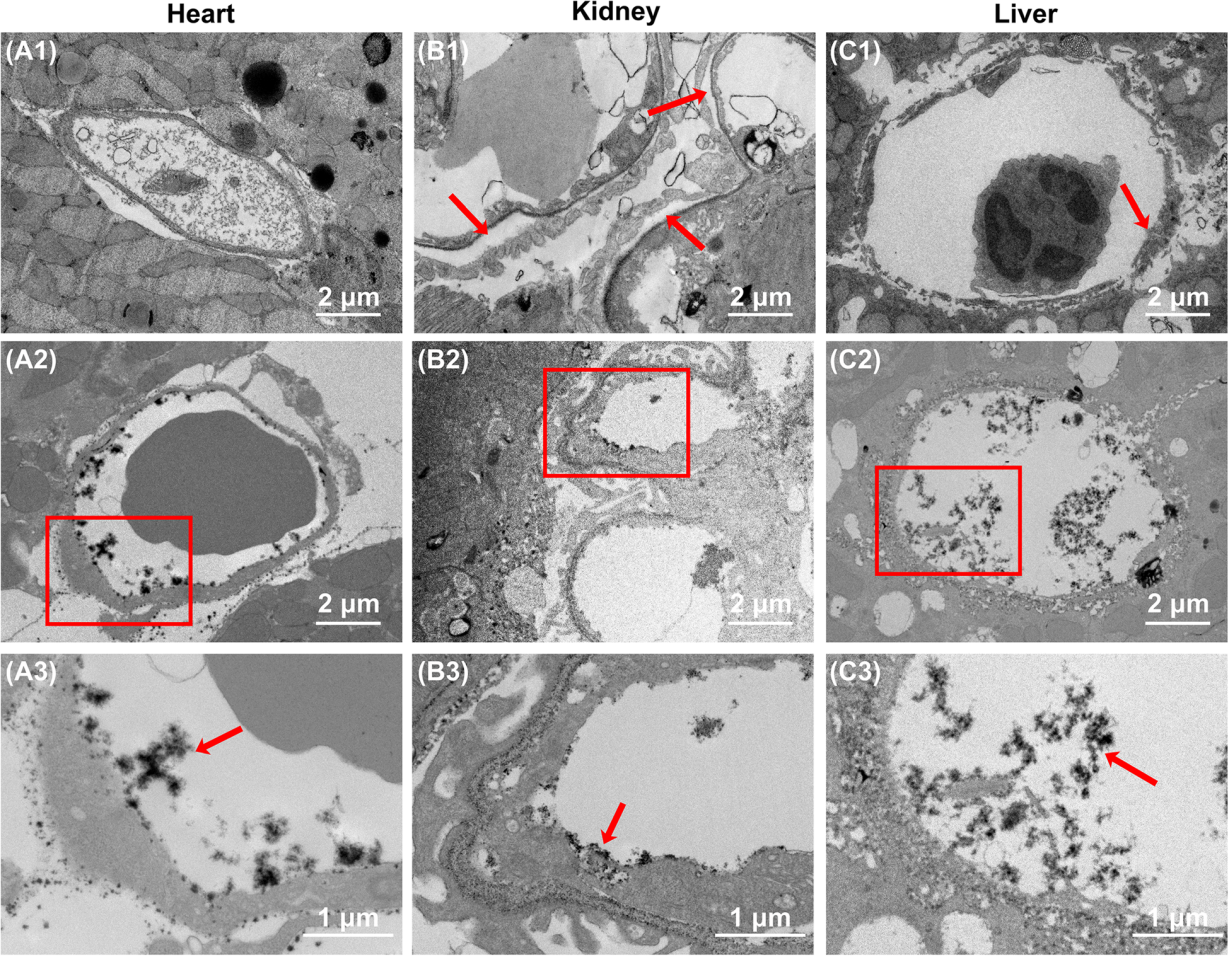
Transmission electron microscopy showing glycocalyx in capillaries under septic conditions. **a** Ultrastructure of cardiac capillaries under septic conditions. **a1** Continuous capillary without lanthanum nitrate staining. The capillary wall appears edematous, and there is fibrin deposited inside the capillary lumen. **a2**, **a3** Lanthanum nitrate staining to visualize the endothelial glycocalyx. **a3** Expanded view of the area within the *red rectangle* in (**a2**). The endothelial glycocalyx is peeled away, and there is little glycocalyx on the endothelial cells (*red arrow*). **b** Ultrastructure of glomerular capillaries under septic conditions. **b1** Glomerular capillary without lanthanum nitrate staining. There is a gap between the podocytes and basement membrane under septic conditions (*red arrows*). **b2**, **b3** Lanthanum nitrate staining to visualize the glycocalyx. **b3** Expanded view of the area within the *red rectangle* in (**b2**). The glycocalyx is cast off from the surface of the glomerular endothelial cells and podocytes. **c** Ultrastructure of hepatic sinusoids under septic conditions. **c1** Sinusoid without lanthanum nitrate staining. Whereas the sinusoid is normally composed of discontinuous flat endothelial cells, here the endothelial cells have become edematous, and the large pores are closed (*red arrow*). **c2**, **c3** Visualized glycocalyx in sinusoids. **c3** Expanded view of the area within the *red rectangle* in (**c2**). The endothelial glycocalyx layer of sinusoids has peeled off, and the space of Disse has become unclear

To produce an experimental endotoxemia model, we intraperitoneally administered 20 mg/kg LPS to 10-week-old C57BL6 male mice. Forty-eight hours after LPS administration, 8 (16%) of the 50 injected mice were still alive (Additional file 2: Figure S2a). Syndecan-1 is the core protein in heparan sulfate proteoglycan, which comprises glycocalyx. Syndecan-1 is released from the endothelium upon injury to the glycocalyx, causing its concentration in the circulation to increase [23]. We found that plasma syndecan-1 levels had reached 7.8 ± 0.9 ng/ml 12 h after LPS injection and 14.4 ± 2.0 ng/ml 24 h after injection. By 48 h after LPS injection, however, plasma syndecan-1 levels had returned to baseline (Additional file 2: Figure S2b). In the heart, LPS injection induced edematous changes to the continuous capillaries, whereby fibrin was deposited inside the capillary lumen. In the LPS-administered mice, the endothelial wall thickness was significantly increased compared with sham mice (sham 101.4 ± 10.1 nm, LPS 285.4 ± 37.7 nm; *p* < 0.05). In addition, the glycocalyx was occasionally peeled from the luminal surface of the capillary to form debris (Figs. 4a and 5a). The percentage of endothelial glycocalyx area in capillaries was significantly decreased under septic conditions compared with sham mice (Additional file 3: Figure S3a). In the kidney, LPS injection broke the three tightly bound layers of the glomerular capillary consisting of the fenestrated endothelial cells, basement membrane, and podocytes. This caused the endothelial pores to become less well defined, and there was a widening of the gap between the basement membrane and the podocytes. The glycocalyx was peeled off and formed a residue within the capillary lumen (Figs. 4b and 5b). The percentage of endothelial glycocalyx area in capillaries was significantly decreased under septic conditions compared with sham mice (Additional file 3: Figure S3b). In the liver, the fenestrations in the sinusoids appeared to be closed by the edematous changes to the endothelial cells 48 h after LPS injection. Endothelial glycocalyx appeared to have been shed into the space of Disse (Figs. 4c and 5c). The percentage of endothelial glycocalyx area in capillaries was significantly decreased under septic conditions compared with sham mice (Additional file 3: Figure S3c).

## Discussion

The endothelial glycocalyx has been particularly difficult to characterize and understand in terms of its three-dimensional structure because of its fragility and instability [1]. Indeed, in addition to trauma, surgery, hyperglycemia, and sepsis, even subtle stimuli such as a plasma volume expansion can disrupt the glycocalyx structure [20, 23–27]. Disruption of glycocalyx exposes the endothelial cells to oxidative damage, and vascular hyperpermeability is observed in sepsis and chronic conditions such as diabetes and hypertension [5, 28]. There has been much effort to visualize endothelial glycocalyx using TEM and substitution of the original ruthenium red staining with lanthanum or alcian blue [7, 29, 30]. In the present study, we adopted lanthanum nitrate staining with a careful perfusion method that entailed (1) incision in the right atrial appendage to relieve pressure during perfusion fixation; (2) neck ligation for better perfusion of the heart, kidney, and liver; and (3) use of a perfusion pump to ensure a steady rate of infusion. With this approach, we were able to successfully observe the three-dimensional ultrastructure of glycocalyx.

Because the structure of endothelial cells can be continuous, fenestrated, or sinusoid, depending on the organ, we anticipated that the structure of endothelial glycocalyx would also vary accordingly. In continuous capillaries in the heart, moss-like endothelial glycocalyx spread over the entire luminal wall of the vessel. By contrast, endothelial glycocalyx in hepatic sinusoids was smaller than in other types of capillaries. An earlier study demonstrated that sinusoidal glycocalyx is substantially smaller than that in pulmonary and cremaster muscle capillaries [31]. The fenestrated capillaries of the renal glomeruli have a full basement membrane, endothelial glycocalyx, and numerous small pores, and a glycocalyx layer lined the open fenestrations. Anatomically, the fenestrations are as much as 65 nm wide, but their effective pore size is only about 15 nm owing to the presence of the glycocalyx [4, 32]. The effective pore size for glomerular filtration beyond the capillary basement membrane is limited to about 6 nm by filtration slit diaphragms at the level of podocyte foot processes. Thus, albumin and larger molecules are not normally filtered into tubular fluid. Sinusoidal endothelial cells express uptake receptors for hyaluronic acid. By actively removing this important glycosaminoglycan, which is a main component of glycocalyx, these cells prevent development of an effective endothelial glycocalyx [4]. Systemic inflammation, such as sepsis, leads to endothelial dysfunction, which in turn increases paracellular permeability and outflow of albumin/fluid into the interstitial space [4]. It is speculated that this effect might be caused by glycocalyx disruption. Previous reports suggested that degradation of endothelial glycocalyx contributes to the pathogenesis of acute respiratory distress syndrome [33, 34]. Similarly, disruption of glycocalyx in cardiac endothelium was observed under septic conditions.

Albuminuria (a reliable marker of sepsis-induced endothelial barrier alterations) is greatly increased in an experimental model of sepsis, presumably in association with changes to the structure of glycocalyx [35]. The extent of glycocalyx injury is estimated indirectly by penetration of red blood cells [36, 37] or serum syndecan-1 concentration [23]. In fact, serum syndecan-1 was used as an endothelial injury marker in recent clinical research [38, 39]. The present study indicated that serum syndecan-1 was increased but endothelial glycocalyx was degraded after LPS administration. These results are consistent with that earlier report, provide clear structural evidence of injured glycocalyx in septic mice, and support that serum syndecan-1 is useful glycocalyx injury marker.

There are currently no clinical therapeutic strategies to treat sepsis through endothelial glycocalyx protection. This is despite compelling evidence that endothelial glycocalyx disruption contributes to the vascular hyperpermeability seen in sepsis. Although corticosteroids decrease the inflammatory damage to the endothelium in systemic sepsis [40], their use in the treatment of sepsis is controversial because systemic glucocorticoid administration raises the likelihood of secondary infection. Antioxidant therapies may help to preserve the integrity of glycocalyx [25], but definitive evidence of the clinical utility of antioxidants in sepsis is still lacking. That said, several previous reports have shown that intact glycocalyx may be protective against endothelial disorders [5, 25, 41, 42]. We therefore suggest that control of endothelial glycocalyx has the potential to mediate a positive therapeutic effect in endothelial disorders.

### Study limitations

Because lanthanum has the capacity to bind with not only glycocalyx but also calcium binding sites, it has been used as a calcium probe in several biological systems [43]. Therefore, it is hard to say that the lanthanum staining technique is specific for only glycocalyx. Likewise, lanthanum nitrate staining for glycocalyx visualization may influence the glycocalyx structure by itself. Because the field able to be observed by electron microscopy is very tiny, the precision of quantification might be limited.

Cardiac output of 10-week-old mice was estimated 16 ± 1.9 ml/minute according to previous reports [44–46]. The perfusion rate of 1 ml/minute is much lower than the flow rate, and we performed incision of the right atrial appendage before perfusion. However, Arkill et al. used a direct pressure transducer to ensure that perfusion pressure of the fluid injected would not affect the glycocalyx layer [47]. Our perfusion method may have challenges because of the lack of such direct pressure measurement.

## Conclusions

In the present study, we visualized the three-dimensional ultrastructure of endothelial glycocalyx in healthy continuous, fenestrated, and sinusoidal capillaries, and we also showed their disruption under experimental endotoxemic conditions. The latter may provide a morphological basis for the microvascular endothelial dysfunction associated with septic injury to organs.

## Additional files

Additional file 1: Figure S1. Backscattered electron images. Backscattered electron image of a renal glomerulus and a sample of liver tissue. **a**–**c** Backscattered electron images of specimens shown in Fig. 2a2, b2, and c2, respectively. Backscattered electrons are detected high-energy electrons from lanthanum. (TIF 26024 kb)

Additional file 2: Figure S2. Profile of septic model mice administered LPS. **a** Survival curves for sham and septic model mice. *p < 0.05 vs. sham. The survival rate is significantly lower in the LPS group than the sham group. **b** Time course of the change in syndecan-1 levels measured by ELISA in plasma from LPS-injected mice. *p < 0.05 vs. before LPS injection. (TIF 17841 kb)

Additional file 3: Figure S3. Percentage of endothelial glycocalyx area in capillaries. The percentage of endothelial glycocalyx area of (**a**) heart, (**b**) kidney, and (**c**) liver capillaries in sham and LPS-injected mice. *p < 0.05 vs. sham. (TIF 25080 kb)

## Abbreviations

C: Carbon
O: Oxygen
La: Lanthanum
LPS: Lipopolysaccharide
P: Phosphorus
S: Silicon
SEM: Scanning electron microscopy
TEM: Transmission electron microscopy
