# Supplementary figures and images for: Three-dimensional ultrastructure of capillary endothelial glycocalyx under normal and experimental endotoxemic conditions
Source: Crit Care. 2017 Oct 23;21:261. doi: 10.1186/s13054-017-1841-8 (PMC5651619; doi:10.1186/s13054-017-1841-8)

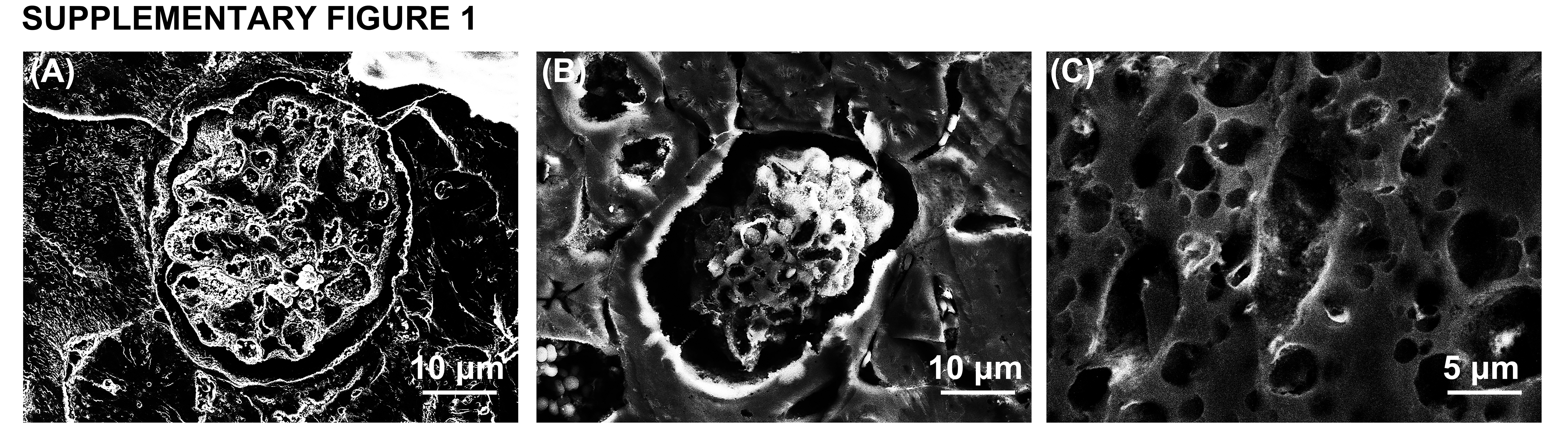

Supplement: Supplementary file 1 — Backscattered electron images. Backscattered electron image of a renal glomerulus and a sample of liver tissue. a–c Backscattered electron images of specimens shown in Fig. 2a2, b2, and c2, respectively. Backscattered electrons are detected high-energy electrons from lanthanum. (TIF 26024 kb) [file 13054_2017_1841_MOESM1_ESM.tif]

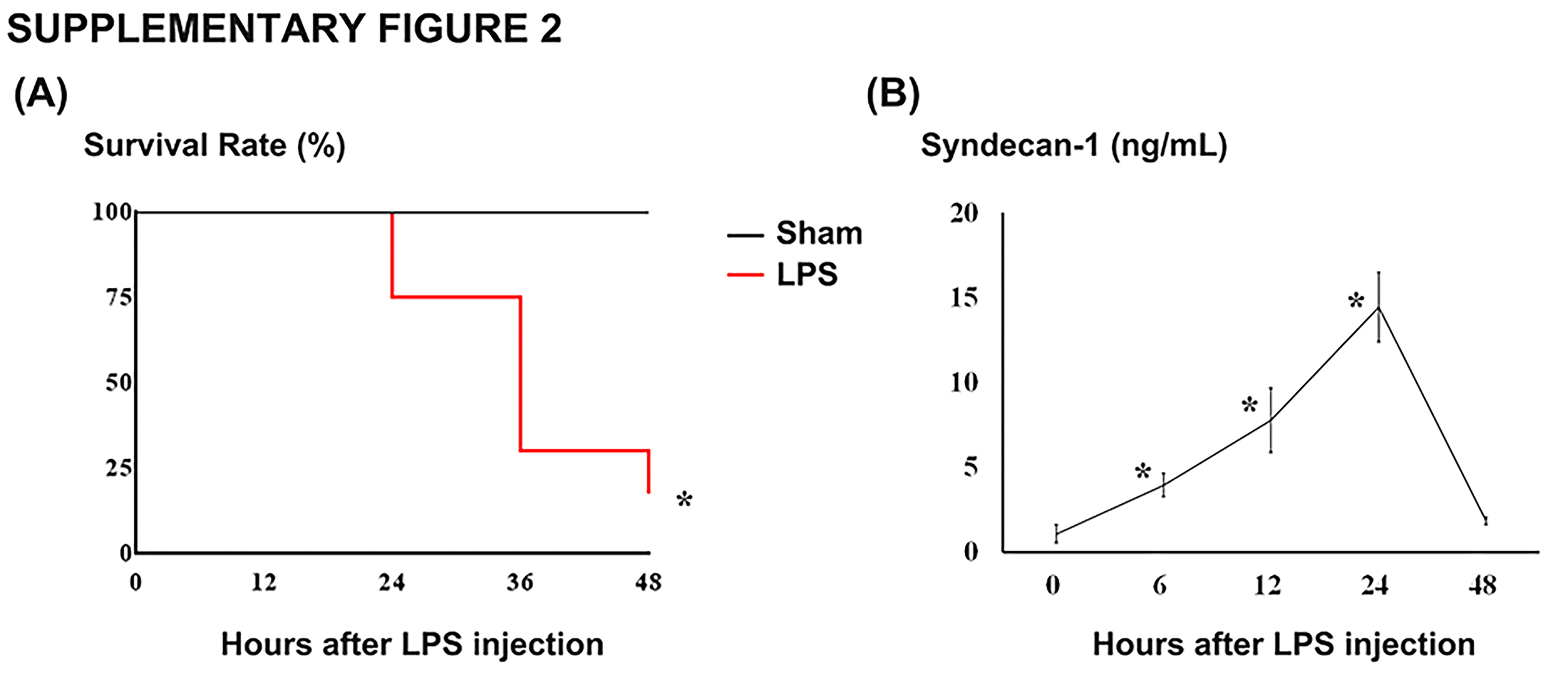

Supplement: Supplementary file 2 — Profile of septic model mice administered LPS. a Survival curves for sham and septic model mice. *p < 0.05 vs. sham. The survival rate is significantly lower in the LPS group than the sham group. b Time course of the change in syndecan-1 levels measured by ELISA in plasma from LPS-injected mice. *p < 0.05 vs. before LPS injection. (TIF 17841 kb) [file 13054_2017_1841_MOESM2_ESM.tif]

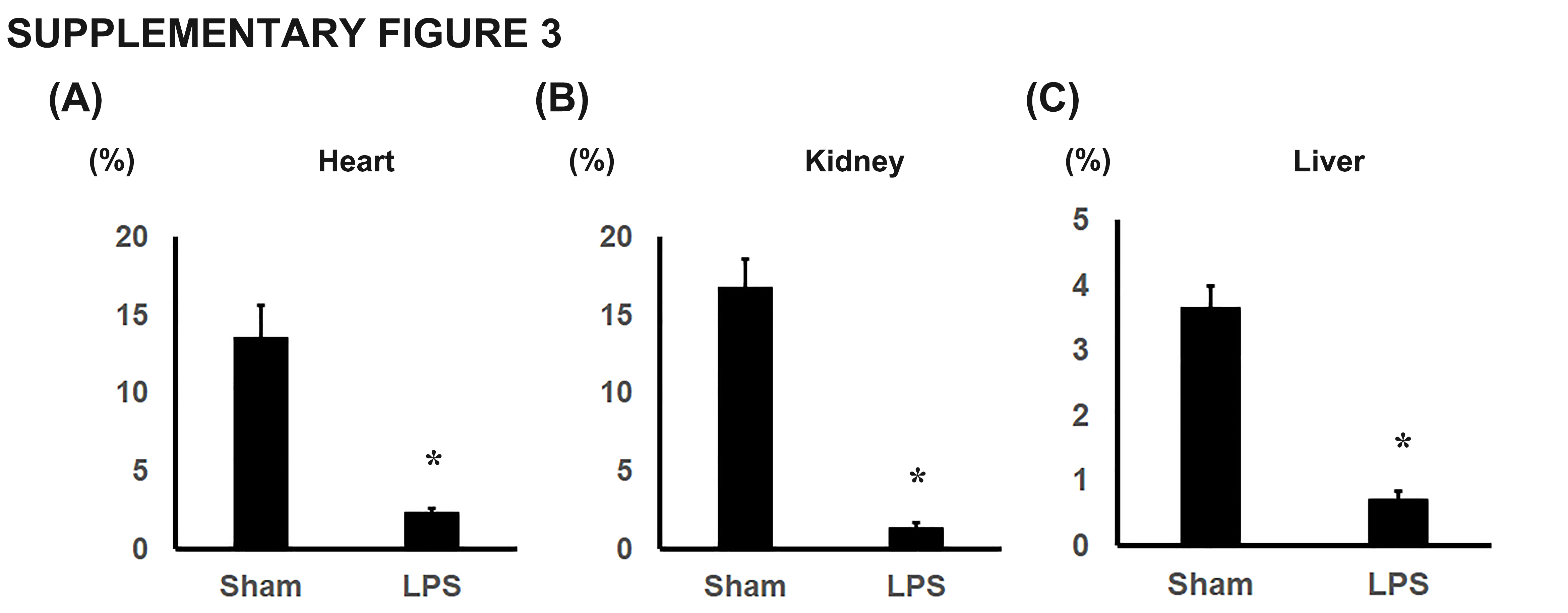

Supplement: Supplementary file 3 — Percentage of endothelial glycocalyx area in capillaries. The percentage of endothelial glycocalyx area of (a) heart, (b) kidney, and (c) liver capillaries in sham and LPS-injected mice. *p < 0.05 vs. sham. (TIF 25080 kb) [file 13054_2017_1841_MOESM3_ESM.tif]
